# Supplementary material for: Long-Term Effectiveness of a Smartphone App and a Smart Band on Arterial Stiffness and Central Hemodynamic Parameters in a Population with Overweight and Obesity (Evident 3 Study): Randomised Controlled Trial
Source: Nutrients. 2022 Nov 10;14(22):4758. doi: 10.3390/nu14224758 (PMC9695348; doi:10.3390/nu14224758)
Supplement: Supplementary file 1 [file nutrients-14-04758-s001.zip › nutrients-1977070-supplementary.pdf]

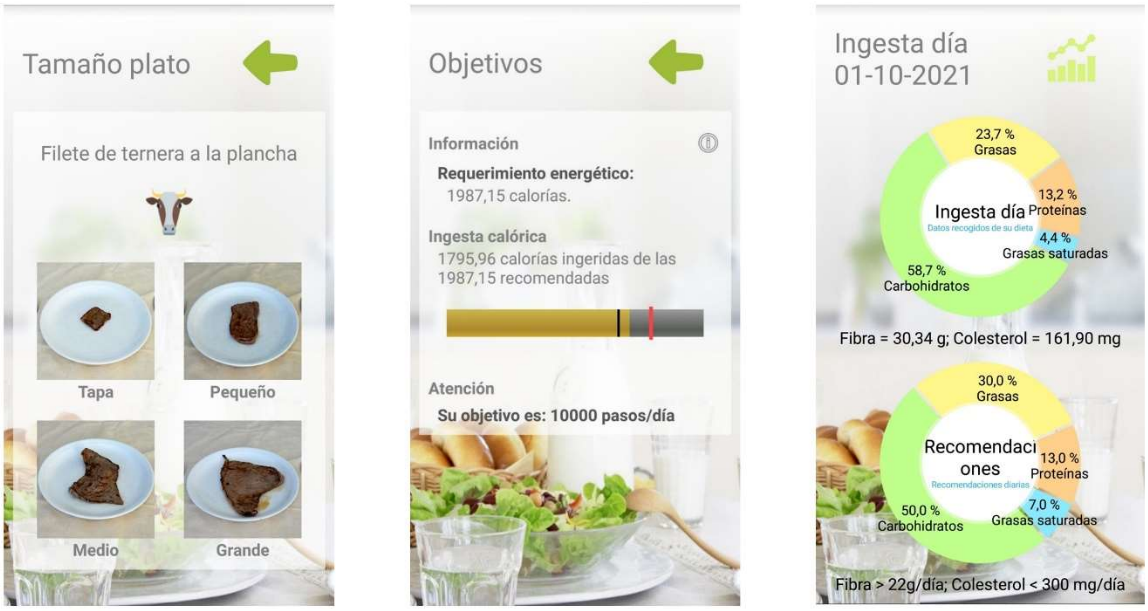


**Figure S1.** Evident 3 app screenshots.

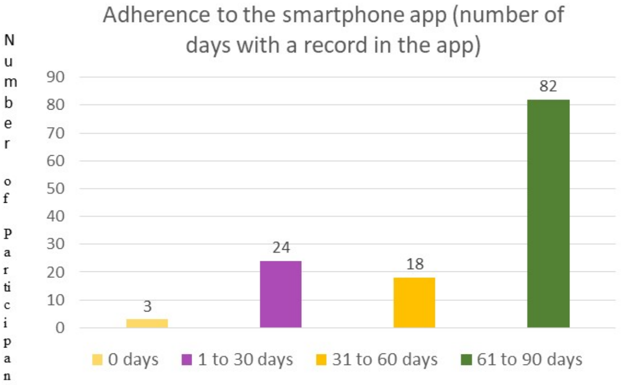


Figure S2. Adherence to the app categories

**Table S1.** Baseline characteristics comparison between subjects who completed the study and those who dropped out.

| Characteristics | Complete (*n* = 217) | Loss of follow-up (*n* = 36) | *p* value |
|---|---|---|---|
| **Age, mean years (SD)** | 47.83 (9.85) | 47.51 (9.40) | 0.813 |
| **Sex, n (%)** | | | 0.564 |
| Men | 66 (30.4) | 11 (30.6) | |
| Women | 151 69.6) | 25 (69.4) | |
| **Smoking status, n (%)** | 87 (40.1) | 11 (30.6) | 0.533 |
| Non smoker | 44 (20.3) | 9 25.0) | |
| Smoker | 86 (39.6) | 16 (44.4) | |
| Former smoker | 87 (40.1) | 11 (30.6) | 0.533 |

| | | | |
|---|---|---|---|
| **Clinical variables, mean (SD)** | | | |
| BMI (kg/m2) | 32.81 (3.24) | 33.17 (3.50) | 0,536 |
| Systolic blood pressure (mmHg) | 115.17 (13.80) | 116.78 (17.26) | 0,534 |
| Diastolic blood pressure (mmHg) | 78.96 (8.56) | 80.60 (11.11) | 0,353 |
| Heart rate (bpm) | 68.13 (9.76) | 71.83 (9.52) | 0,056 |
| Total Cholesterol (mg/dL) | 193. 77 (34.21) | 186.89 (30.26) | 0,263 |
| HDL Cholesterol (mg/dL) | 50.69 (12.10) | 53.00 (11.90) | 0,294 |
| LDL Cholesterol (mg/dL) | 118.10 (29.36) | 11.00 (27.27) | 0,182 |
| Triglicerides (mg/dL) | 123.29 (63.84) | 122.31 (91.68) | 0,938 |
| Glycaemia (mg/dL) | 90.76 (15.32) | 94.34 (20.91 | 0,226 |
| HbA1c (%) | 5.47 (0.41) | 5.52 (0.65) | 0,541 |
| Cardiovascular Risk (%) | 6.01 (5.81) | 5.78 (7.44) | 0,833 |
| **Chronic diseases, n (%)** | | | |
| Hypertension | 57 (26.3) | 11 (30.6) | 0.362 |
| Dyslipidemia | 48 (22.1) | 6 (17.1) | 0.338 |
| Diabetes Mellitus | 3 ((1.4) | 1 (2.8) | 0.461 |
| **Medication use, n(%)** | | | |
| Antihypertensive drugs | 36 (16.6) | 4 (11.1) | 0.288 |
| Lipid-lowering drugs | 36 (16.6) | 5 (13.9) | 0.451 |

BMI, body mass index; bmp, beats per minute; LDL–cholesterol, low-density lipoprotein cholesterol; HDL cholesterol, high-density lipoprotein cholesterol; HbA1c, glycosylated hemoglobin. Categorical variables are expressed as n (%) and continuous variables as mean ± standard deviation. * Statistically significant differences ($p < 0.05$).

**Table S2.** Correlation between days of use of the app and measures analyzed globally and by sex.

| | Global | Men | Women |
|---|---|---|---|
| 3-month change, mean difference | | | |
| cfPWV, m/sec | -0.106 | 0.006 | -0.154 |
| baPWV, m/sec | -.180* | -0.050 | -.287** |
| CAVI | -0.036 | 0.011 | -0.054 |
| cSBP (mmHg) | -0.114 | -0.163 | -0.098 |
| cDBP (mmHG) | -.206* | -0.239 | -0.193 |
| CAIx | -0.023 | 0.154 | -0.097 |
| PAIx | -0.017 | 0.194 | -0.075 |
| AP (mmHg) | 0.067 | 0.135 | 0.055 |
| ED (%) | -0.087 | -0.232 | -0.029 |
| SEVR (%) | 0.161 | 0.188 | 0.156 |
| 12-month change, mean difference | | | |
| cfPWV, m/sec | -0.054 | 0.159 | -0.180 |
| baPWV, m/sec | -0.100 | -0.054 | -0.131 |
| CAVI | -0.019 | -0.036 | -0.013 |
| cSBP (mmHg) | -0.147 | -0.145 | -0.148 |
| cDBP (mmHG) | -0.164 | -0.235 | -0.130 |
| CAIx | -0.079 | 0.148 | -0.169 |
| PAIx | 0.067 | 0.162 | -0.048 |
| AP (mmHg) | -0.048 | 0.267 | -0.140 |
| ED (%) | 0.008 | -0.066 | 0.034 |
| SEVR (%) | -0.048 | -0.024 | -0.050 |

cfPWV, carotid-femoral pulse wave velocity; baPWV, brachial-ankle pulse wave velocity;CAVI, cardio-ankle vascular index. cSBP, central systolic

blood pressure; cDBP, central diastolic blood pressure; CAIx, Central Augmentation Index; PAIx, Peripheral Augmentation Index; AP, Augmented pressure; ED%, ejection duration ratio; SEVR%, subendocardial viability ratio.
